# Ablation of *EYS* in zebrafish causes mislocalisation of outer segment proteins, F-actin disruption and cone-rod dystrophy

**DOI:** 10.1038/srep46098

**Published:** 2017-04-05

**Authors:** Zhaojing Lu, Xuebin Hu, Fei Liu, Dinesh C. Soares, Xiliang Liu, Shanshan Yu, Meng Gao, Shanshan Han, Yayun Qin, Chang Li, Tao Jiang, Daji Luo, An-Yuan Guo, Zhaohui Tang, Mugen Liu

**Affiliations:** 1 Key Laboratory of Molecular Biophysics of Ministry of Education, Department of Genetics and Developmental Biology, College of Life Science and Technology, Huazhong University of Science and Technology, Wuhan, Hubei, 430074, P.R China; 2 MRC Human Genetics Unit/Centre for Genomic & Experimental Medicine, MRC Institute of Genetics and Molecular Medicine, University of Edinburgh, Edinburgh, EH4 2XU, United Kingdom; 3 Department of Medical Genetics, School of Basic Medical Sciences, Wuhan University, Wuhan, Hubei, P.R China; 4 Department of Bioinformatics and Systems Biology, College of Life Science and Technology, Huazhong University of Science and Technology, Wuhan, Hubei, 430074, P.R China

## Abstract

Mutations in *EYS* are associated with autosomal recessive retinitis pigmentosa (arRP) and autosomal recessive cone-rod dystrophy (arCRD) however, the function of EYS and the molecular mechanisms of how these mutations cause retinal degeneration are still unclear. Because *EYS* is absent in mouse and rat, and the structure of the retina differs substantially between humans and *Drosophila*, we utilised zebrafish as a model organism to study the function of *EYS* in the retina. We constructed an *EYS*-knockout zebrafish-line by TALEN technology which showed visual impairment at an early age, while the histological and immunofluorescence assays indicated the presence of progressive retinal degeneration with a cone predominately affected pattern. These phenotypes recapitulate the clinical manifestations of arCRD patients. Furthermore, the *EYS*^−/−^ zebrafish also showed mislocalisation of certain outer segment proteins (rhodopsin, opn1lw, opn1sw1, GNB3 and PRPH2), and disruption of actin filaments in photoreceptors. Protein mislocalisation may, therefore, disrupt the function of cones and rods in these zebrafish and cause photoreceptor death. Collectively, these results point to a novel role for EYS in maintaining the morphological structure of F-actin and in protein transport, loss of this function might be the trigger for the resultant cellular events that ultimately lead to photoreceptor death.

Hereditary retinal degeneration is a group of heterogeneous diseases, which is a major cause of human visual impairment characterised by the progressive dysfunction and death of retinal photoreceptors^1^. To date, about 200 retinal degeneration genes have been identified (https://sph.uth.tmc.edu/retnet/). A role for *Eyes shut homolog (EYS*, OMIM 612424) in retinal degeneration was first identified in 2008^2^. *EYS*, which spans more than 2 Mb within the *RP25* locus (6q12), consists of 43 exons and encodes a 3165 amino acid protein localised in the outer segment of the photoreceptor^2^,^3^. Mutations in *EYS* are recognised as a major cause for autosomal recessive retinitis pigmentosa (arRP)^4^,^5^,^6^,^7^, accounting for 5–18% arRP patients in different populations^4^,^8^,^9^, and have also been identified in patients with autosomal recessive cone-rod dystrophy (arCRD)^10^,^11^. So far, about 100 *EYS* mutations, predominantly truncation mutations along with some missense mutations have been reported^2^,^3^,^4^,^5^,^6^,^7^,^8^,^9^,^10^,^11^,^12^,^13^,^14^,^15^,^16^,^17^. Remarkably, there are no mutation hot spots within *EYS*, and these mutations are scattered throughout the gene.

The human EYS protein starts with a signal peptide and contains 21 epidermal growth factor (EGF)-like domains in its N-terminus followed by five C-terminal LamG domains, interspersed among additional EGF repeats^2^. Its orthologous protein in *Drosophila,* spacemaker (SPAM), is secreted by photoreceptor cells and is essential for the formation of the matrix-filled inter-rhabdomeral space^18^,^19^. The EYS protein is therefore suggested to play a role in the modelling of retinal architecture in the human eye^2^. Apart from SPAM in *Drosophila,* EYS homologues have been noted in several other species (for example, zebrafish, chicken, platypus, horse, dog), while EYS is absent in some other species such as insects with a closed rhabdom system, or in some mammals like mouse and rat^4^.

Previous studies on *EYS* have utilised *Drosophila* as a model organism, but there still remain unresolved questions owing to the different retinal structure between *Drosophila* and human^20^,^21^. Notably, *Drosophila* possesses a compound eye, however, humans lack this compound eye structure. Because zebrafish has a similar retinal structure and a cone-dominant vision like in humans^22^,^23^, and is a widely used animal model in biological research^24^,^25^,^26^, it confers advantages to better interpret the pathological and molecular mechanisms of *EYS* mutations. Very recently, an independent study with *EYS*-knockout zebrafish was reported by Yu *et al*.^27^. Their results showed retinal degeneration with a cone-predominately affected pattern in the *EYS*-knockout zebrafish. Their data also revealed that EYS is located near the connecting cilium, and is important for the normal shape of the ciliary pocket and for survival of photoreceptors. However, the precise function of *EYS* in the photoreceptor and the mechanism of how *EYS* mutations lead to retinal degeneration are still unclear.

In this study, we generated an *EYS* knockout zebrafish line using TALEN (transcription activator-like effector nuclease) technology. In the *EYS*-knockout (*EYS*^−/−^) zebrafish, we observed, retinal function was affected at an early age post-fertilisation, while the thickness of the outer nuclear layer (ONL) of the retina decreased over time, with progressive cone and rod degeneration. Additionally, we also detected mislocalisation of certain photoreceptor outer segment (OS) proteins (rhodopsin, opn1lw, opn1sw1, GNB3, and PRPH2), as well as an abnormal morphology of actin filaments (F-actin). Our findings reveal a progressive retinal degeneration in *EYS*^−/−^ zebrafish and suggest a novel role of EYS in maintaining the morphological structure of F-actin and in protein transport, which could enhance our understanding of *EYS*-deficient pathogenesis and the function of EYS.

## Results

### Generation of *EYS*^−/−^ zebrafish lines by TALEN

**Figure 1:**
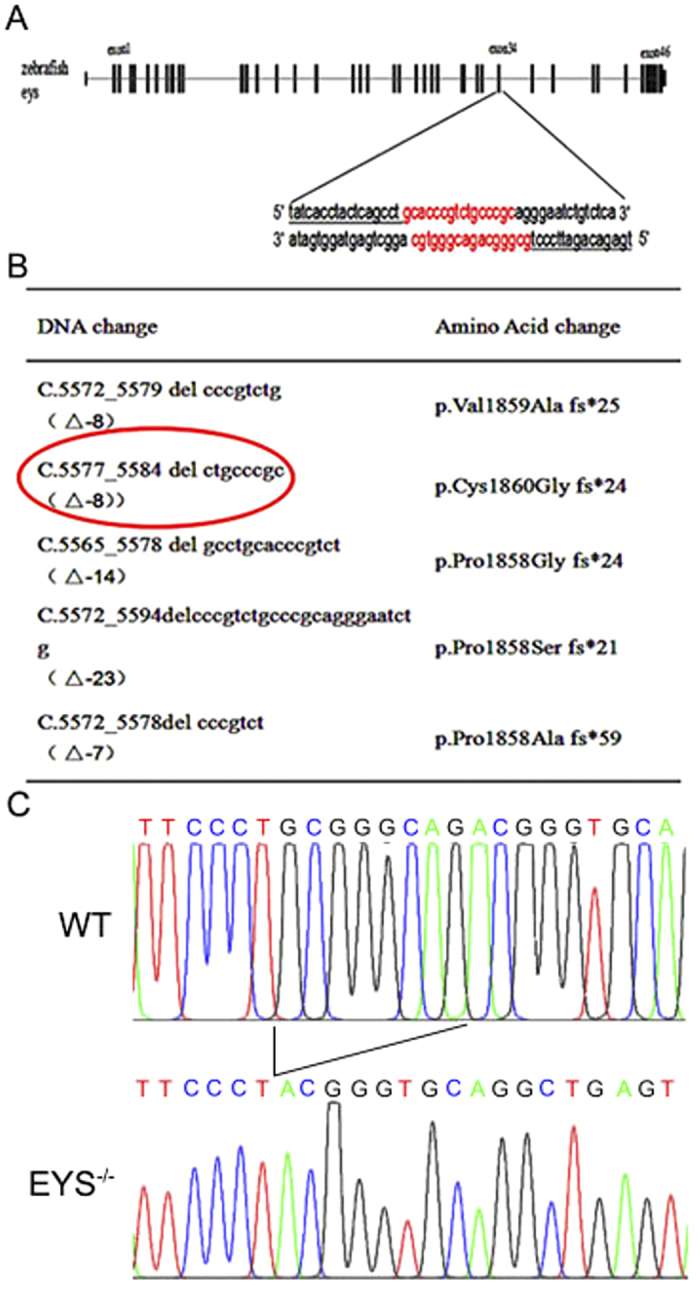
Generation of the *EYS* knockout zebrafish. (**A**) The 47 exons of zebrafish *EYS* gene is shown with the left and right arms of the TALE binding sequences underlined. (**B**) Genomic sequences of *EYS* mutations. The c.5577_5584delCTGCCCGC (del8) *EYS* mutation is indicated with a red oval. (**C**) Sequencing validation of the del8 homozygous zebrafish.

The zebrafish *EYS* genomic sequences (NC_007124.6) and cDNA sequences were downloaded from the NCBI database. TALENs of left and right binding sites were separated by a 16-bp DNA spacer (Fig. 1A). By mutant screening over three generations of zebrafish, we identified five types of mutations (Fig. 1B), and all of these mutant-types caused frameshift, and truncated EYS proteins. We selected one of these mutations (c.5577_5584delCTGCCCGC, named del8) that led to a premature termination of the encoded protein, for subsequent experiments. Homozygous del8 zebrafish were identified by restriction endonuclease AciI digestion analysis, and the genomic DNA sequencing results are shown in Fig. 1C. The mutated cDNA sequences including a section of the region encompassing exon 34 and exon 35 are shown in Supplementary Figure 1, that demonstrate the knockout design was effective. We failed to generate a usable antibody by immunising rabbits with the zebrafish EYS protein fragment. Although we also tried to use the commercial antibody anti-human EYS from Novus Biological (Cat# NBP1-90038, which is used in the reported paper^27^), it did not work well in our study (Supplementary Figure 2A,B in the revision).

### *EYS* ^−/−^ zebrafish display impaired retinal visual function and reduced expression of phototransduction cascade genes at an early age

**Figure 2:**
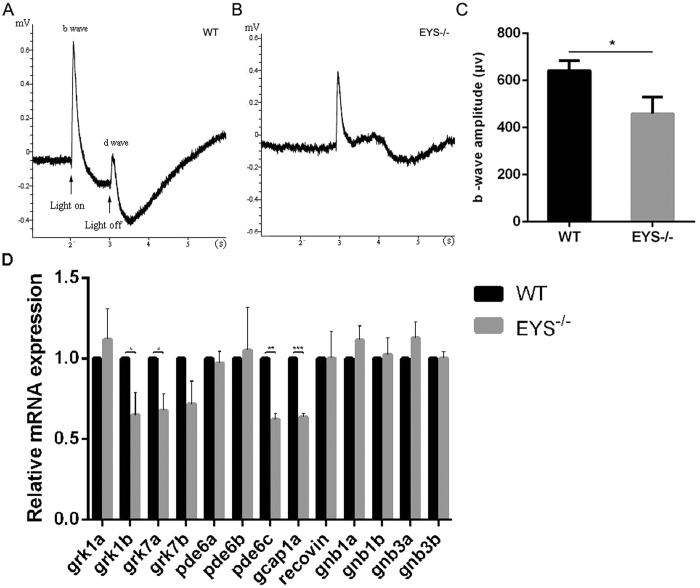
Visual impairment in the *EYS*^−/−^ zebrafish detected by ERG analysis. (**A**) Detection of visual function by ERG analysis of WT at 10 dpf. The arrows indicate the start and the end of light stimulation, respectively. (**B**) Detection of visual function by ERG analysis of *EYS*^−/−^ zebrafish at 10 dpf. (**C**) Comparison of b-wave amplitudes between WT and *EYS*^−/−^ zebrafish (n = 5); P = 0.0116. (**D**) Quantitative real-time PCR analysis of phototransduction cascade genes at 10 dpf. Gapdh was served as endogenous control. Data from three independent experiments revealed a significant reduction of the expression of grk1b (P = 0.0491), grk7a (P = 0.0337), pde6c (P = 0.0029), gcap1a (P = 0.0008) in the *EYS*^−/−^ zebrafish.

To assess the visual function of the retina, we performed electroretinography (ERG) experiments at 10 dpf (days-post-fertilisation) for WT and *EYS*^−/−^ zebrafish. The results showed that the scotopic b-wave amplitudes of *EYS*^−/−^ zebrafish were reduced by about 28% compared with WT zebrafish (Fig. 2A–C), suggesting that in the *EYS*^−/−^ zebrafish, vision loss originated at an early age.

Phototransduction response starts when the photoreceptors sense light and is followed by G protein-mediated signalling processes. The impaired retinal visual function indicated by ERG prompted us to examine the expression of the phototransduction cascade genes including rod-special genes (grk1a/b, pde6a/b, gnb1a/b), cone-special genes (grk7a/b, pde6c, gnb3a/b), and both photoreceptors-containing genes (gcap1a, recoverin). Results from quantitative real-time PCR showed significant decrease in expression of grk1b, grk7a, pde6c and gcap1a at 10 dpf (Fig. 2D). These results further demonstrated that the visual function was affected in the *EYS*-deficient zebrafish at an early age.

### *EYS* ^−/−^ zebrafish show a progressive degeneration of ONL

**Figure 3:**
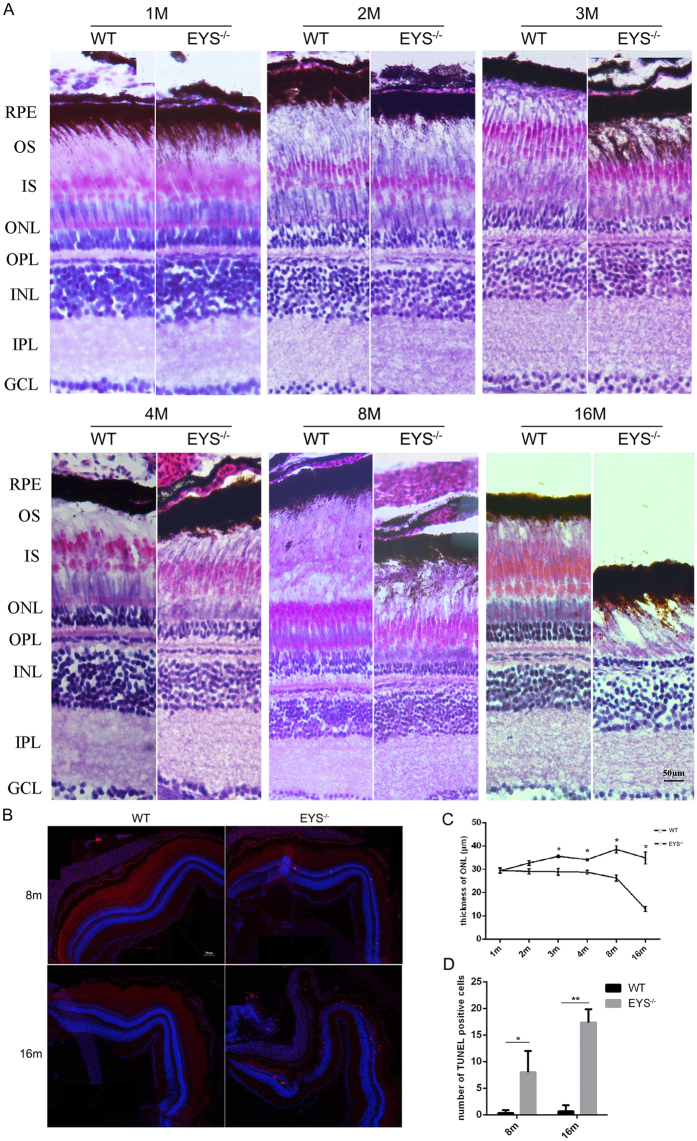
The thickness of ONL decreased and the apoptosis of retinal cells increased in the *EYS*^−/−^ zebrafish. (**A**) Retinal histology analysis of WT and *EYS*^−/−^ zebrafish. Retinal sections of *EYS*^−/−^ zebrafish stained with hematoxylin/eosin (HE) at indicated ages. RPE, retinal pigment epithelium; OS, outer segment; IS, inner segment; ONL, outer nuclear layer; OPL, outer plexiform layer; INL, inner nuclear layer; IPL, inner plexiform layer; GCL, ganglion cell layer. Scale bars: 50 μm. (**B**) TUNEL staining between WT and *EYS*^−/−^ zebrafish. Scale bars: 50 μm. (**C**) Comparison of the ONL thickness between WT and *EYS*^−/−^ zebrafish at indicated ages (n = 3). Quantitative analysis revealed a significant reduction of the ONL thickness at 3 mpf (P = 0.0479), 4 mpf (P = 0.0258), 8 mpf (P = 0.0122), 16 mpf (P = 0.0272) in the *EYS*^−/−^ zebrafish. (**D**) Quantification of TUNEL positive cells in WT and *EYS*^−/−^ zebrafish at indicated ages (n = 3). Quantitative analysis revealed a significant increase of TUNEL positive cells at 8 mpf (P = 0.0341), 16 mpf (P = 0.0028) in the *EYS*^−/−^ zebrafish.

Next, we examined the retinal morphology of the WT and *EYS*^−/−^ zebrafish. Retinal sections of *EYS*^−/−^ zebrafish obtained at different months stained with hematoxylin/eosin (HE) did not show obvious morphological differences in cell layer organisation. However, the thickness of the ONL in *EYS*^−/−^ zebrafish showed a significant decrease over time (Fig. 3A). At the age of 1 month post-fertilisation (mpf), no difference in thickness of the ONL was observed between WT and *EYS*^−/−^ zebrafish, but at 2 mpf a decrease was apparent in *EYS*^−/−^ zebrafish, while it significantly decreased to about 81.5% of that in WT zebrafish by 3 mpf, and to ~37.4% of WT zebrafish when 16 mpf (Fig. 3C). The whole-retina sections with HE staining at 16 mpf are shown in Supplementary Figure 3.

Because a reduction of ONL thickness was observed, we wanted to establish whether this altered status of the *EYS*^−/−^ zebrafish retina was also reflected in an increase in cell death. The levels of apoptosis were therefore analysed by TUNEL (TdT-mediated dUTP nick end labelling) staining on retinal sections (Fig. 3B). Increased levels of apoptotic cells (about 8-fold) were clearly observed in the *EYS*^−/−^ vs. WT zebrafish retina at 8 mpf and it increased 18-fold at 16 mpf (Fig. 3D). However, no positive signal was detected at 2 mpf (Supplementary Figure 4). These data indicate that apoptosis increased gradually in the retina of *EYS*^−/−^ zebrafish.

**Figure 4:**
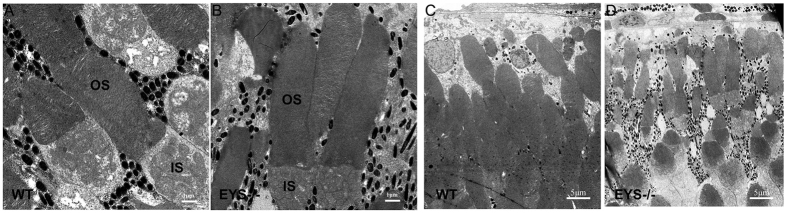
Ultrastructural analysis of the WT and *EYS*^−/−^ zebrafish photoreceptors at 3 mpf. The morphological and disk-stacking of OS is normal between the WT and *EYS*^−/−^ zebrafish (**A**,**B**). The number of photoreceptors in *EYS*^−/−^ zebrafish is less than WT zebrafish (**C**,**D**). Scale bars: 1 μm in (**A**,**B**); 5 μm in (**C**,**D**).

As EYS has been suggested to play a role in the modelling of retinal architecture^2^,^3^, we undertook a transmission electron microscopy (TEM) assay to assess ultrastructural changes of the photoreceptor in *EYS*^−/−^ zebrafish. We found that the morphological and disk-stacking of photoreceptor OS did not exhibit differences in *EYS*^−/−^ zebrafish compared with WT at 3 mpf (Fig. 4A,B); even at 10 mpf, this was normal in *EYS*^−/−^ zebrafish (Supplementary Figure 5). However, we found that the number of photoreceptors in *EYS*^−/−^ zebrafish was less than in WT zebrafish (Fig. 4C,D).

### *EYS* ^−/−^ zebrafish exhibit cone-rod dystrophy

**Figure 5:**
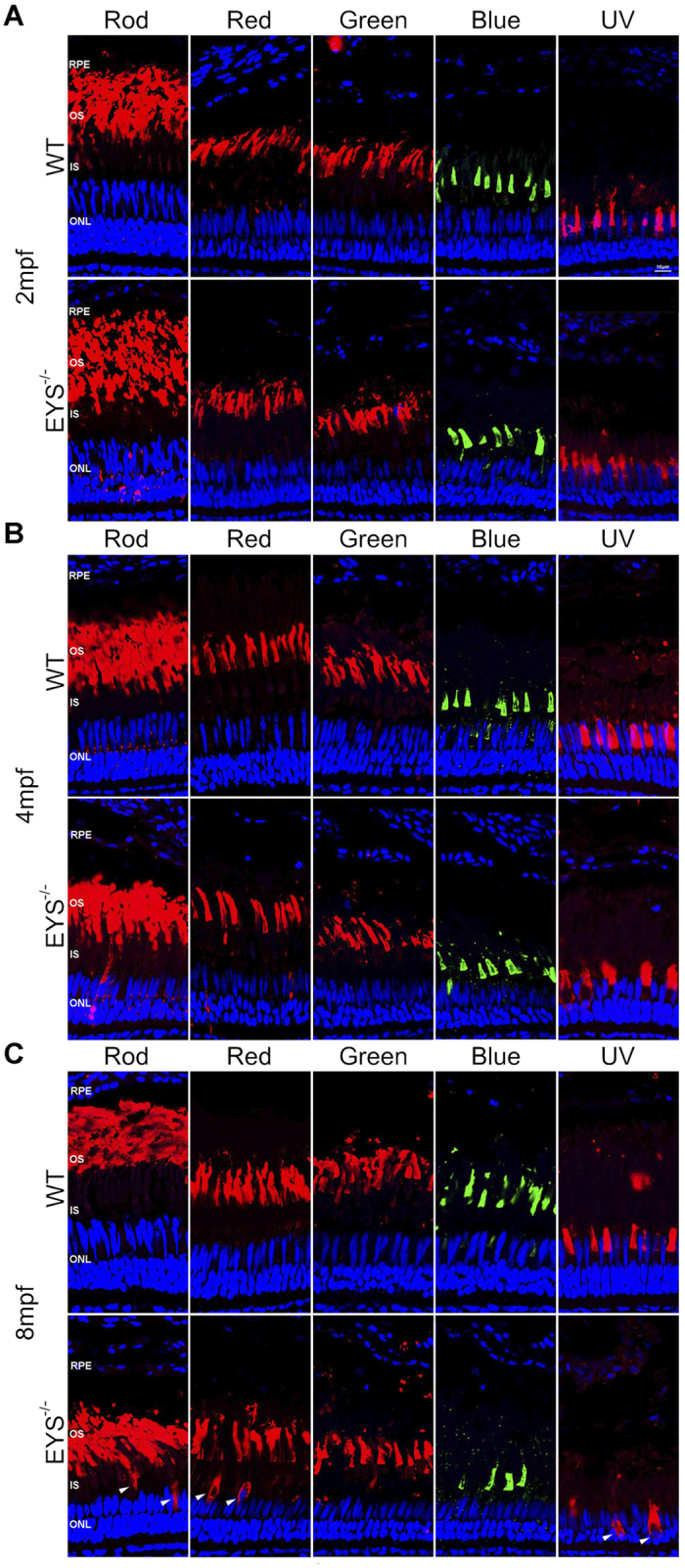
Photoreceptor outer segment is affected in *EYS*^−/−^ zebrafish. Retinal cryosections from WT and *EYS*^−/−^ zebrafish were labelled rods and cones (red, green, blue and UV cone) with specific antibodies at the ages of 2 (**A**), 4(**B**) and 8(**C**) mpf. White arrows indicate the mislocalised OS proteins. Scale bars: 10 μm.

**Figure 6:**
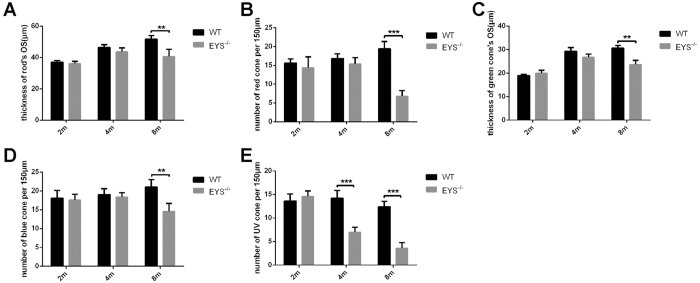
Statistical data for rods and cones (n = 5). (**A**) The rod OS thickness showed a significant reduction at 8 mpf (P = 0.0012); (**B**) the number of red cones decreased at 8 mpf (P < 0.0001); (**C**) the OS thickness of green cones decreased at 8 mpf (P = 0.0048); (**D**) the number of blue cones decreased at 8 mpf (P = 0.0069); (**E**) the number of UV cones decreased at 4 mpf (P = 0.0006), 8 mpf (P = 0.0004).

Zebrafish possess similar photoreceptor types to human, except for the UV (ultra-violet) cone^23^. To explore possible differences in these photoreceptors, we labelled rods and cones (red, green, blue and UV cone) with specific antibodies (rhodopsin, opn1lw, opn1mw, opn1sw2 and opn1sw1) in retinal sections for WT and *EYS*^−/−^ zebrafish, at different ages. We found that the number of rods in *EYS*^−/−^ retina did not differ from WT (Fig. 5A–C), but the length of rods OS progressively decreased; it decreased to 79% of WT zebrafish by age of 8 mpf (Fig. 6A). Next, we tested the OS of the four types of cones. The number of red and UV cones OS in *EYS*^−/−^ retinas exhibited a significant decrease over time. At 8 mpf, the number of red cones OS decreased to 35% of WT zebrafish (Fig. 6B), while the number of UV cones OS decreased to 49% of WT zebrafish by 4 mpf (Fig. 6E). In contrast, the number of green cones OS did not show an obvious reduction between the WT and *EYS*^−/−^ zebrafish retina, but the length of green cones OS underwent progressive reduction, and decreased to 77% at 8 mpf compared with WT retina (Fig. 6C). However, the number of blue cones decreased to 70% at 8 mpf compared with WT retina (Fig. 6D; the whole retinal cryosections, labelled with rods and cones at 16 mpf, are shown in Supplementary Figure 6).

### *EYS* ^−/−^ zebrafish show mislocalisation of photoreceptor OS proteins

**Figure 7:**
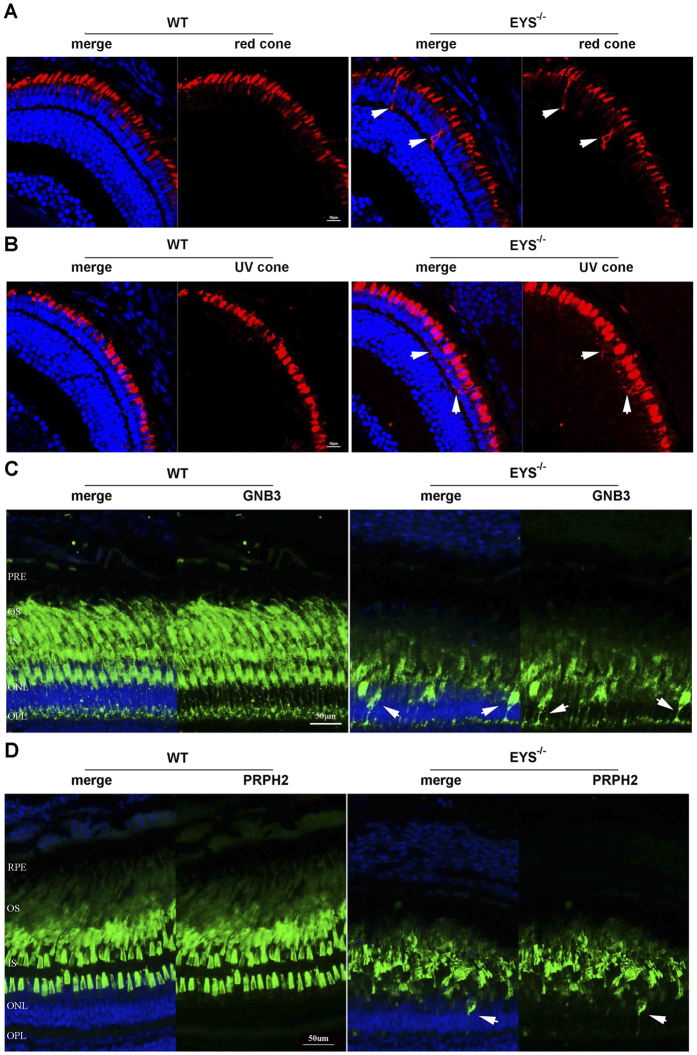
Photoreceptor OS proteins were mislocalised in *EYS*^−/−^ zebrafish. (**A**) Retinal cryosections from WT and *EYS*^−/−^ zebrafish were immunostained with anti-opn1LW antibodies at 10 dpf. White arrows indicated the mislocalised opn1LW proteins. Scale bars: 10 μm. (**B**) Retinal cryosections from WT and *EYS*^−/−^ zebrafish were immunostained with anti-opn1SW1 antibodies at 10 dpf. White arrows indicated the mislocalised opn1SW1 proteins. Scale bars: 10 μm. (**C**) Retinal cryosections from WT and *EYS*^−/−^ zebrafish were immunostained with anti-GNB3 antibodies at the age of 5 mpf. White arrows indicate the mislocalised GNB3 proteins. Scale bars: 50 μm. (**D**) Retinal cryosections from WT and *EYS*^−/−^ zebrafish were immunostained with anti-PRPH2 antibodies at the age of 7 mpf. White arrows indicated the mislocalised PRPH2 proteins. Scale bars: 50 μm.

In addition to the reduction of photoreceptor OS numbers and length, we also found some opsin proteins were mislocalised in the *EYS*^−/−^ zebrafish retina. The red cones opsin, UV cones opsin, and rhodopsin were mislocalised to the IS (inner segment) and synapse of the photoreceptor (Fig. 5C). Slight mislocalisation of red and UV cones opsin proteins were initially detected at the age of 10 dpf (Fig. 7A,B), but with time, substantial mislocalisation was observed. To ascertain if this mislocalisation was only limited to opsin proteins, we investigated two other OS proteins, the protein G-protein beta subunit (GNB3) and the peripherin2 (PRPH2). GNB3 is a membrane protein located on the cone OS and plays a role in phototransduction^28^, while PRPH2 is assumed to be essential for disk membrane morphogenesis and renewal both in rods and cones^29^,^30^. Similar results were obtained after immunofluorescence labelling with antibodies against the two proteins; both proteins showed decreased fluorescence signal and mislocalised to IS in *EYS*^−/−^ zebrafish retina (Fig. 7C,D; the western blot results of these two proteins are shown in the Supplementary Figure 7). Moreover, the arrangement of PRPH2 was irregular compared to the parallel arrangement in WT retina. Because mislocalisation of OS-associated proteins was detected at the age of 10 dpf, when the thickness of ONL, the number of photoreceptors, and the apoptosis of retinal cells did not show significant changes, we conclude it was the major reason leading to photoreceptor degeneration in *EYS*^−/−^ zebrafish retina.

### The integrity of F-actin is disrupted in *EYS* ^−/−^ zebrafish

**Figure 8:**
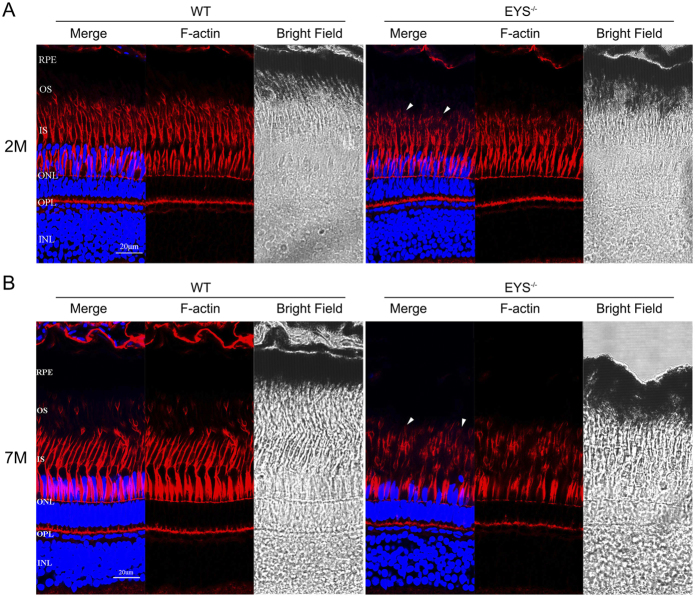
F-actin disruption in *EYS*^−/−^ zebrafish. Retinal cryosections were immunostained with phalloidin between WT and *EYS*^−/−^ zebrafish. (**A**) F-actin was disrupted, slightly, in *EYS*^−/−^ zebrafish at 2 mpf. (**B**) F-actin was disrupted, substantially, in *EYS*^−/−^ zebrafish at 7 mpf. White arrows indicate the disrupted F-actin. Scale bars: 20 μm.

Previous studies reported that the actin cytoskeleton had specific effects on the translocation of arrestin, transducin and cyclic nucleotide-gated channel α-subunit (CNGA1)^31^,^32^, which indicated a crucial role for F-actin in protein transport. As mislocalisation of OS proteins was observed, using phalloidin staining (sigma) we next examined F-actin to assess whether the actin cytoskeleton morphology was affected in the retina of *EYS*^−/−^ zebrafish. In WT zebrafish retina, F-actin exhibited a characteristic organisation, with long cables of polymerised F-actin arranged in parallel to the major axis of the photoreceptor cell. On the other hand, the *EYS*^−/−^ zebrafish retina showed slightly reduced and disrupted F-actin at 2 mpf (Fig. 8A), but was severely disrupted at 7 mpf (Fig. 8B). Although the morphological structure of F-actin showed no apparent change in WT and *EYS*^−/−^ zebrafish retina at 10 dpf, it could be the result of less affected photoreceptors that are not easily observed (Supplementary Figure 8).

## Discussion

*EYS* mutations are associated with arRP and arCRD, both of these retinal diseases lead to photoreceptor death and vision loss. In order to understand the pathological process and molecular mechanism of retinal degeneration caused by *EYS* mutations for better clinical diagnosis and therapy, we generated an *EYS* knockout zebrafish-line, identified progressive retinal degeneration in this *EYS* deficient model and investigated the function of zebrafish *EYS in vivo*.

The onset ages of retinal degeneration caused by *EYS* mutations in patients range from 6 to 62 years of age^4^,^8^. Most patients show a diminished light response in ERG recordings at an early age^11^,^33^. Retinal histopathology evaluation in EYS mutant donor eyes from patients with arRP shows very thin ONL with few photoreceptors left on the retina^34^. Likewise, our *EYS*^−/−^ zebrafish showed an early reduction in ERG b-wave recordings, likely resulting from the reduced mRNA expression of photoreceptor phototransduction cascade genes (grk1b, grk7a, pde6c, and gcap1a). Meanwhile, the ONL thickness of the *EYS*^−/−^ zebrafish retina showed a progressive decrease from the third month, until it disappeared. This phenotype is consistent with the general characteristics of progressive retinal degeneration, described previously^11^,^33^,^34^. The results of the TEM assay also indicate photoreceptor impairment in *EYS*^−/−^ zebrafish. Although the morphology of OS and disk stacking were normal, the numbers of photoreceptor were clearly decreased in the *EYS*^−/−^ zebrafish retina. Furthermore, the detection of apoptosis in the *EYS*^−/−^ zebrafish retina could help explain the changes of these photoreceptors. Similar results of apoptosis have also been found in many retinal degeneration diseases^35^,^36^,^37^, where apoptosis caused the loss of photoreceptors.

arRP and arCRD patients present with similar clinical features during the middle and late periods, so it is critical to understand the pathological process early on for the accurate clinical diagnosis. Using our *EYS*^−/−^ zebrafish models, we found that the five types of photoreceptors exhibited different means of degeneration. Overall, the cones degenerated earlier than the rods. These results also similar with the recent report by Yu *et al*., in their study the *EYS*-knockout zebrafish also showed retinal degeneration with a cone-predominately affected pattern^27^. This phenotype recapitulates the clinical manifestations of arCRD, which may be a helpful reference for clinical diagnosis in patients with *EYS* mutations.

Opsin is the primary visual pigment in the photoreceptor and is restricted to the OS compartment^23^. In some animal models of retinal degeneration (for example, RPE65, RPGR, RP2 and CC2D2A knockout models), opsin proteins are often mislocalised throughout the cellular components and consequently photoreceptor death ensues^38^,^39^,^40^,^41^. In our study, we also observed mislocalisation of opsin proteins (rhodopsin, opn1lw, opn1sw1) in the *EYS*^−/−^ zebrafish retina. At the age of 10 dpf we detected slight mislocalisation of opn1lw and opn1sw1, and with time, the number of photoreceptors containing mislocalised opsin proteins, increased. Furthermore, we showed two other OS locus proteins GNB3 and PRPH2 were mislocalised too. These results indicate that mislocalisation is not limited to opsin proteins, but could be a wider trend, which could be a key reason for photoreceptor death.

Actin filament bundles in photoreceptors extend from the inner segment and terminate in the calycal processes, which surrounded the proximal outer segment in rods^42^, and are paraxially aligned with the outer segment in cones^43^. In our study, we observed the actin fibre bundle in OS or the calycal processes are disrupted from the second month onwards in the *EYS*^−/−^ zebrafish retina photoreceptor, which indicated the calycal processes were affected. As the photoreceptor calycal processes have a role in increasing the rigidity of the inner-outer segment junction^44^ and F-actin plays a crucial role in protein transport, we speculated that disorganisation of F-actin after *EYS* deficiency in zebrafish may be associated with OS proteins mislocalisation. Meanwhile, as calycal processes are present in human and zebrafish retina, but is absent in mouse^45^, also indicates an underlying role of *EYS* in the modelling of calycal processes. Further research is necessary to elucidate the specific relationship between EYS and F-actin.

In summary, by using TALEN technology we generated an *EYS*^−/−^ zebrafish line. By analysis of the physiological and pathological progression from 10 dpf to 16 mpf, we concluded that loss of *EYS* in zebrafish mainly leads to cone and rod dystrophy, and our detailed description of the pathological processes in *EYS*^−/−^ zebrafish would be useful references for clinical diagnosis. Finally, the observations of disorganised F-actin and mislocalised OS proteins suggest important roles for EYS in the photoreceptor, which provides insight into possible molecular mechanisms of photoreceptor death caused by *EYS*-mutation and establishes a solid foundation for further mechanistic studies.

## Methods

### Zebrafish maintenance

All procedures of the animal experiments were reviewed and approved by the Institutional Animal Care and Use Committee at the College of Life Science and Technology, Huazhong University of Science and Technology and all experiments were conducted according to the relevant guidelines. Zebrafish larvae and adult were maintained at 26–28.5 °C under a 14/10 hour light/dark cycle. Fertilised eggs were collected and maintained in E3 medium in an incubator (at ~28.5 °C) for 72 hours until the larvae hatched.

### Antibodies

List of antibodies used in the present study is provided in Supplementary Table 1.

### Generation of *EYS* ^−/−^ zebrafish

The *EYS* knockout zebrafish line was constructed by utilising the Golden Gate TALEN kit, and the sequence-specific TAL effector repeats were chosen by using the web server https://boglab.plp.iastate.edu/. TALEN mRNAs were synthesised and purified by the T3 mMessage mMachine Kit (Ambion, Austin, TX, USA). A pair of left and right TALEN mRNAs was mixed at a ratio of 1:1 and then microinjected into one-cell stage eggs of wild-type zebrafish. 48 h after mRNAs injection, 10–20 embryos were collected from the total and a mixed genomic DNA extracted. A 455 bp DNA fragment containing the *EYS* target site was amplified by PCR, using the flowing primers: Forward primer: tgcctggataaactgcgatagat and Reverse primer: ggaggattttggcagtgatgtag. The efficiency of the TALEN-mediated mutagenesis was determined by the restriction enzyme AciI digestion analysis. The rest of the embryos were raised to sexual maturity (3 months) and outcrossed with wild-type zebrafish to get the first generation heterozygous mutant zebrafish. The F1 zebrafish genotype also was determined by PCR and sequencing, then the same zebrafish genotype was selected to get the second generation, which contained the homozygote mutant zebrafish.

### Hematoxylin-Eosin staining

Zebrafish eyes were isolated and fixed with 4% paraformaldehyde in PBS for 12 h at 4 °C, cryoprotected in 30% sucrose overnight, and embedded in OCT compound. Cryostat sections (10–15 μm thick) containing the whole retina including the optic disk was stained with hematoxylin and eosin. For each section, digitised images of the retina were captured using a commercial imaging system. The thickness of the outer nuclear layer (ONL) was plotted versus distance (100 μm) from the optic nerve head. Three zebrafish from each group were included in this analysis. The thickness of *EYS* mutant ONL was compared with that of same age wild-type.

### Transmission electron microscopy

Zebrafish eyes were isolated and left in the fixative (2.5% glutaraldehyde in 0.1 M PBS buffer, pH 7.4) overnight at 4 °C. After three washes with PBS, the eyes were fixed in 1% osmium tetroxide for 2 hours at RT then dehydrated through an ethanol gradient followed by treatment with propylene oxide and embedded in epoxy medium. Ultrathin sections of 100 nm thickness were prepared using an ultramicrotome and stained for transmission electron microscopy (TEM).

### Electroretinography

Protocols for the zebrafish larvae ERG recordings were described previously^46^. In brief, after 30 minutes of dark adaption, zebrafish larvae at 10 dpf were paralysed with Esmeron (0.8 mg/mL in E3 medium; MedChem Express). The larvae were then placed on a wet filter paper over the reference electrode and the recording electrode put on the centre of the cornea. ERGs were recorded after 5 minutes complete dark adaption. A 1-second single stimulus was used to make a typical ERG trace. All the traces were collected within 20 minutes late afternoon, and the average of the top five b-wave amplitudes was regarded as the larva’s b-wave amplitude.

### Immunofluorescence

For immunofluorescence staining, cryosections were rinsed with PDT (PBS solution containing 1% DMSO and 0.1% Triton X-100) for 10 min and blocked with blocking solution (PDT containing 1% BSA and 10% normal goat serum) for 1 h at RT. Primary antibodies were prepared in blocking solution containing 2% normal goat serum and slides were incubated overnight at 4 °C. Slides were washed 3 times with PDT and incubated with Alexa Fluor 488 nm or 594 nm secondary antibody (1:1000; Molecular Probes^®^) for 1 h at 37 °C. DAPI was diluted with PBS to final 5 ug/mL and used to label the nucleus. The slides were washed 3 times with PBS and then mounted under glass coverslips. Fluorescence images were captured using a confocal laser-scanning microscope (FluoView^TM^ FV1000 confocal microscope, Olympus Imaging).

### TUNEL staining

TUNEL staining was performed using the TUNEL BrightRed Apoptosis Detection Kit (Vazyme Biotech) according to the manufacturer’s instructions. Generally, cryosections were air-dried at RT, fixed with 4% paraformaldehyde in PBS for 30 minutes. The slides were washed 2 times with PBS for 15 minutes and incubated with proteinase K buffer for 10 minutes. After that, slides were washed 2–3 times with PBS and incubated with equilibration buffer for 10–30 minutes. Then, the retinal sections were incubated in TdT buffer at 4 °C overnight. The next day, after DAPI label, the slides were mounted under glass coverslips.

### RNA extraction and RT-qPCR

Total RNA of zebrafish was extracted using Trizol (Takara), and quantitated by NanoDrop spectrometry (Thermo Scientific, Wilmington, DE, USA). cDNA was generated by MMLV reverse transcriptase (Invitrogen). Real-time PCR was performed using AceQ^®^ qPCR SYBR^®^ Green Master Mix (Vazyme) according to the manufacturer’s instructions and relative gene expression was quantified using the StepOnePlusTM Real-Time PCR System (Life Technologies). Gene primers are listed in Supplementary Table 2.

### Western blot

Zebrafish eyes were isolated and homogenised in cold RIPA lysis buffer with protease inhibitor cocktail. Protein concentration was determined using the BCA protein assay kit (Beyotime, China). Proteins were separated on SDS-PAGE and transferred to nitrocellulose membranes. The membranes were blocked for 2 h at room temperature (RT) in 5% skimmed milk dissolved in TBST buffer, and then incubated with the dilute solution of primary antibodies (Supplementary Table S1) overnight at 4 °C with gentle agitation. After washing in TBST, the membranes were incubated with HRP-conjugated secondary antibodies (1:20000; Thermo) for 2 h at RT. The membranes were then developed using SuperSignal^®^ELISA Femto Maximum sensitivity Substrate (Thermo) and ChemiDoc XRS+ imaging system (Bio-Rad laboratories).

### Statistical analysis

All data are presented as mean ± SD. Data groups were compared by two-tailed Student’s *t*-test using the GraphPad Software. Differences between groups were considered statistically significant if P < 0.05. The statistical significance is denoted by asterisks (*P < 0.05; **P < 0.01; ***P < 0.001).

## Additional Information

**How to cite this article:** Lu, Z. *et al*. Ablation of *EYS* in zebrafish causes mislocalisation of outer segment proteins, F-actin disruption and cone-rod dystrophy. *Sci. Rep.* **7**, 46098; doi: 10.1038/srep46098 (2017).

**Publisher's note:** Springer Nature remains neutral with regard to jurisdictional claims in published maps and institutional affiliations.

## Supplementary Material

Supplementary Data

Supplementary Information
