# Supplementary material for: Ablation of EYS in zebrafish causes mislocalisation of outer segment proteins, F-actin disruption and cone-rod dystrophy
Source: Sci Rep. 2017 Apr 5;7:46098. doi: 10.1038/srep46098 (PMC5380955; doi:10.1038/srep46098)
Supplement: Supplementary Data [file srep46098-s1.pdf]

Supplementary Data

Ablation of *EYS* in zebrafish causes mislocalisation of outer segment proteins, F-actin disruption and cone-rod dystrophy

Zhaojing Lu[1,+], Xuebin Hu[1,+], Fei Liu[1], Dinesh C. Soares[2], Xiliang Liu[1], Shanshan Yu[1], Meng Gao[1], Shanshan Han[1], Yayun Qin[1], Chang Li[1], Tao Jiang[1], Daji Luo[3], An-Yuan Guo[4], Zhaohui Tang[1*], Mugen Liu[1*]

Supplementary Figures

Supplementary Figure 1. cDNA sequencing validation of the del8 in homozygous zebrafish. The cDNA sequences including a section of the region encompassing exon 34 and exon 35, the 8-bp deletion was located in the exon 35 and indicated with a red bracket. (The flowing primers were used: forward primer: tgggcctggagtcaatttcacatg and reverse primer: cgcggcagtagagaagagaaccat).

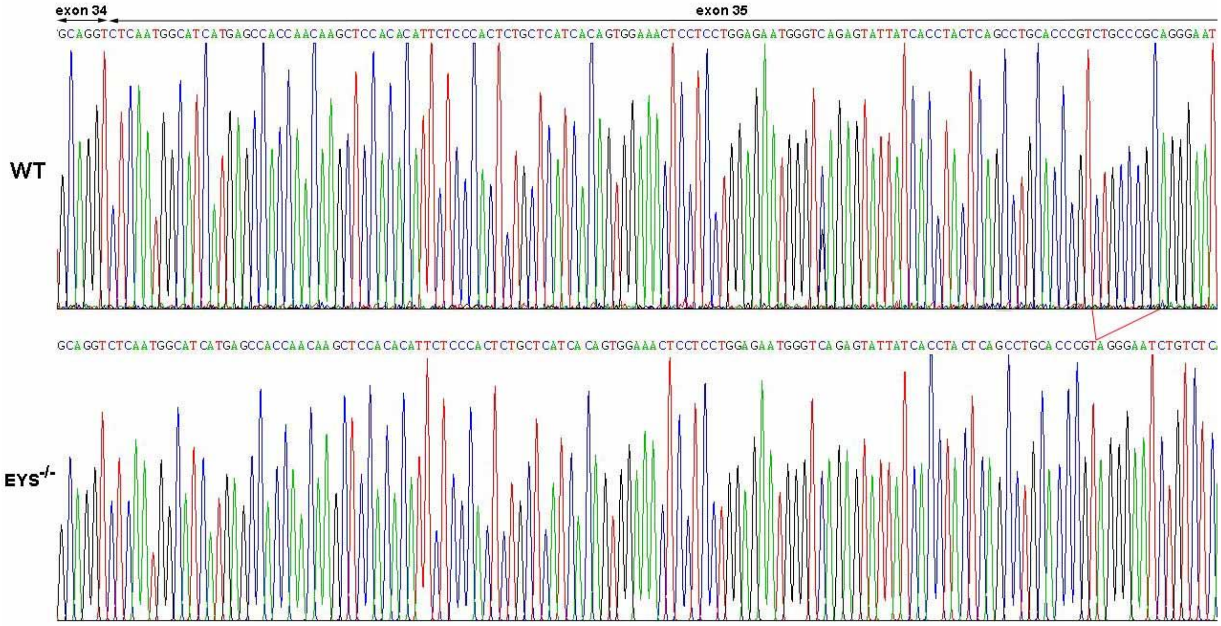

Supplementary Figure 2. Immunostaining of the WT and *EYS*$^{-/-}$ zebrafish retinas at 3mpf using the EYS antibody from Novus Biological (Cat# NBP1-90038, which is used in the reported open bio. paper) (1:300) A and (1:100) B. White arrows indicate the possible positive signal, it was not only found between the retinal pigment epithelium (RPE) and ONL but also located in the ONL and inner nuclear layer (INL). Scale bars: 20μm.

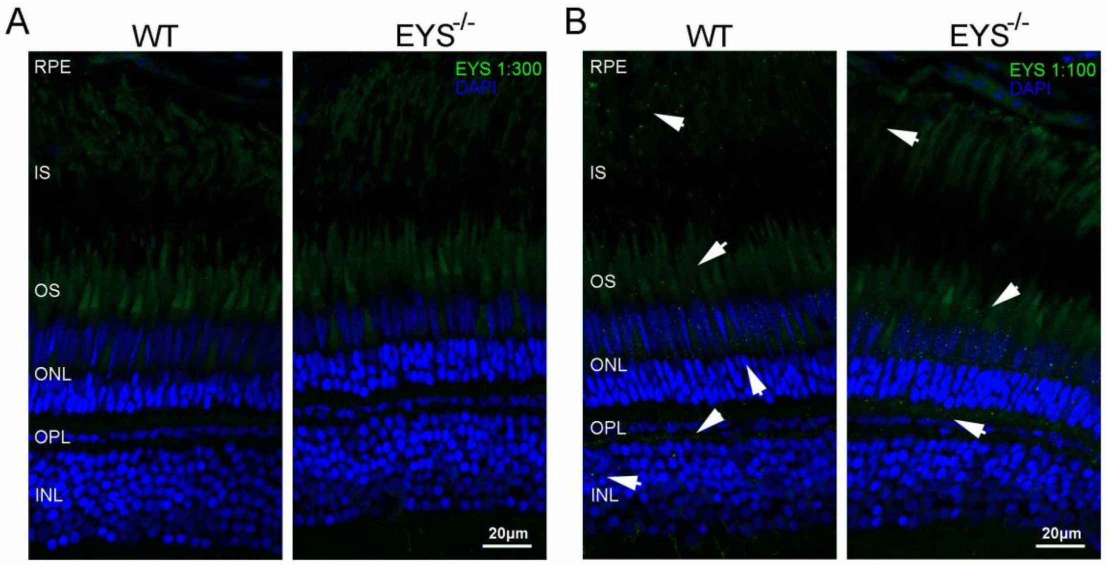

Supplementary Figure 3. The whole retina sections with HE staining at the age of 16mpf. Scale bars: 100μm.

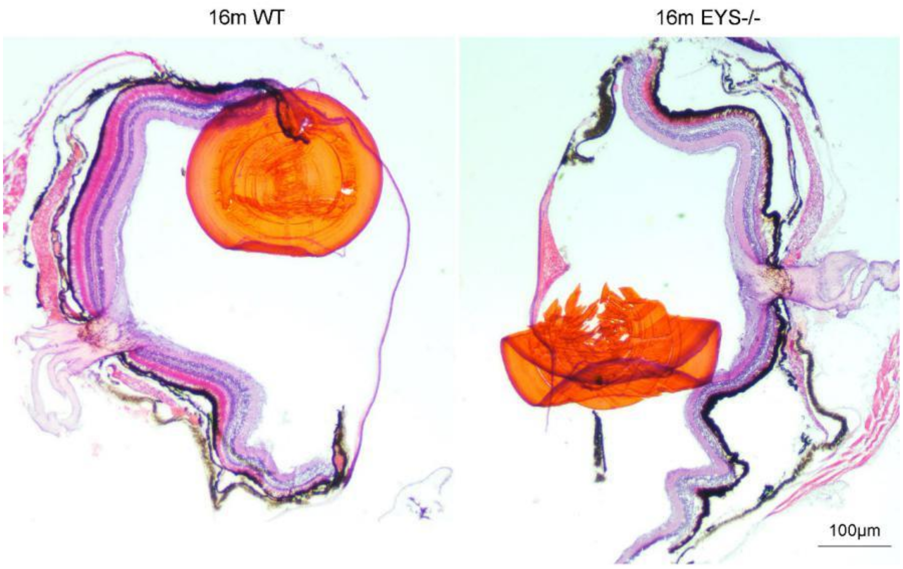

Supplementary Figure 4. TUNEL analysis of WT and *EYS*$^{-/-}$ zebrafish retina at 2mpf. Scale bars: 50μm.

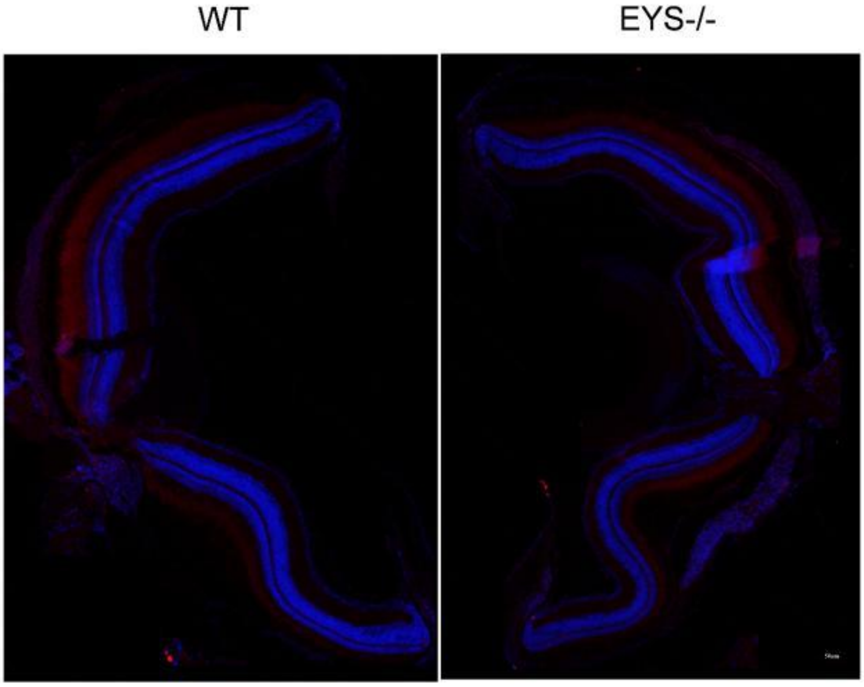

Supplementary Figure 5. Retinal ultrastructural of WT and *EYS*$^{-/-}$ zebrafish at 10mpf. Scale bars: 5μm.

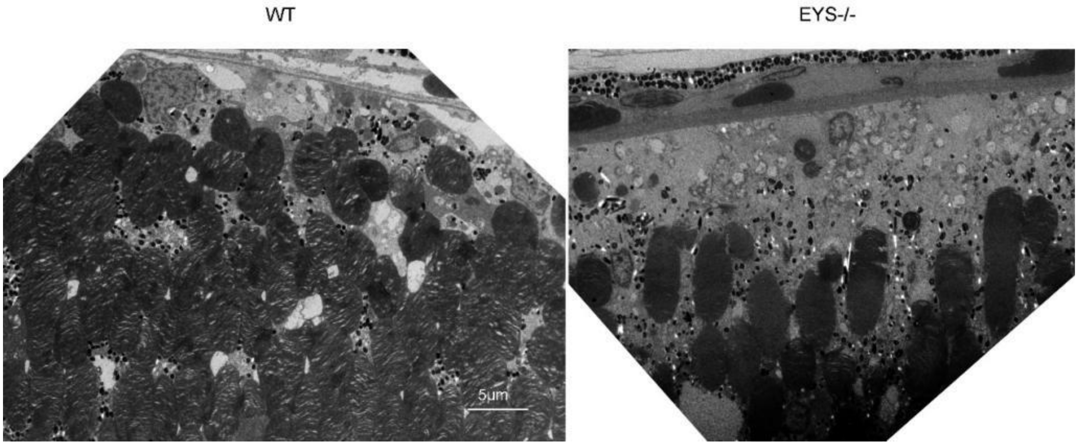

Supplementary Figure 6. The whole retina sections of immunofluorescence analysis with different opsin antibodies at the age of 16mpf. Scale bars: 50μm.

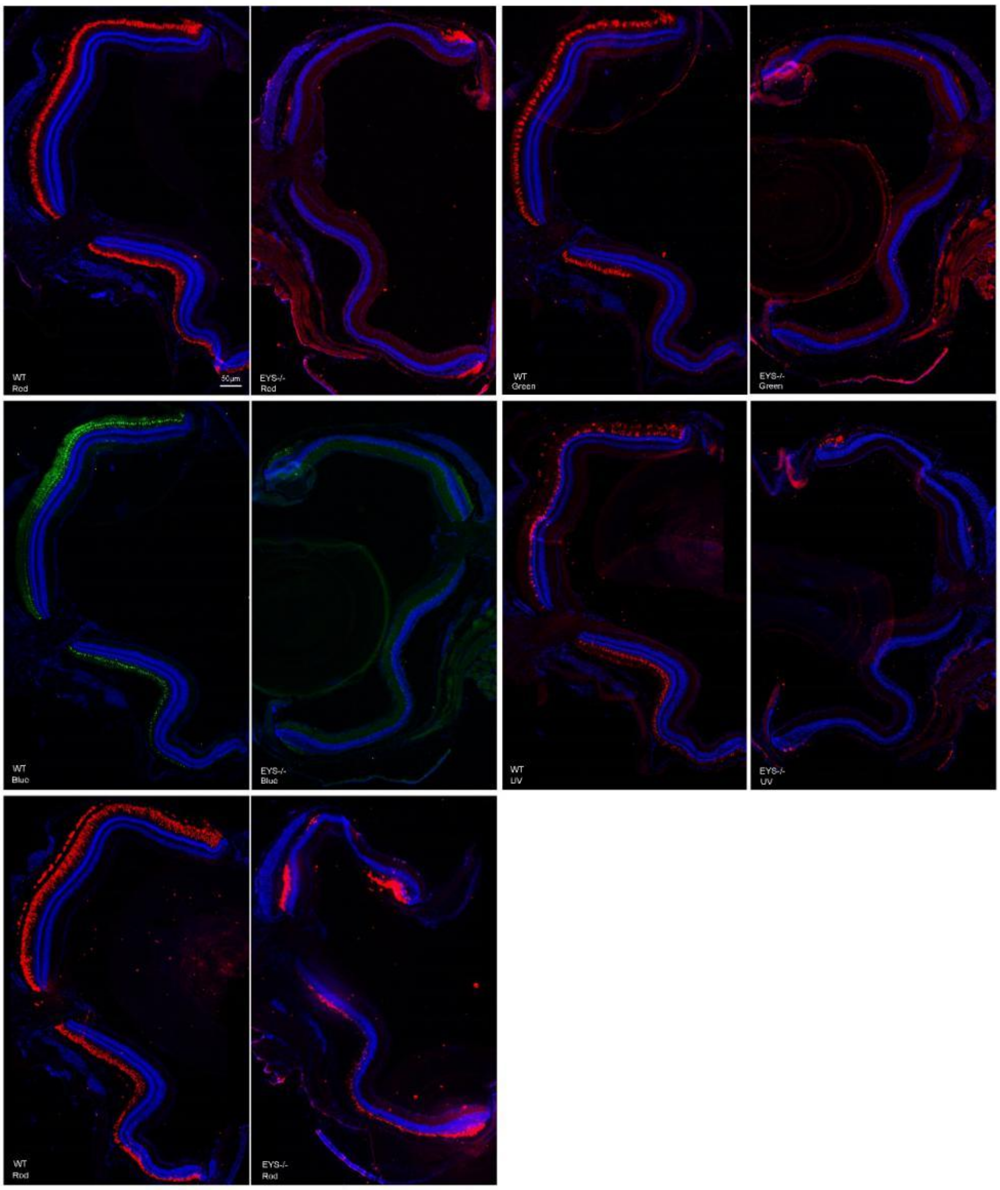

Supplementary Figure 7. Protein levels of GNB3 and PRPH2 were detected by western blot at 6mpf between WT and *EYS*$^{-/-}$ zebrafish (n=5). Alpha tubulin was used as loading control. Quantitative analysis revealed a significant reduction of GNB3 (P=0.0099) and PRPH2 (P=0.0111) in the *EYS*$^{-/-}$ zebrafish. (Full-length gels and blots are shown in the original data of western blot.)

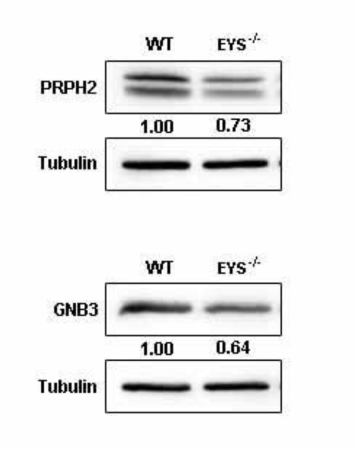

Supplementary Figure 8. Immunostaining of the WT and *EYS*$^{-/-}$ zebrafish retinas at 10dpf with phalloidin.

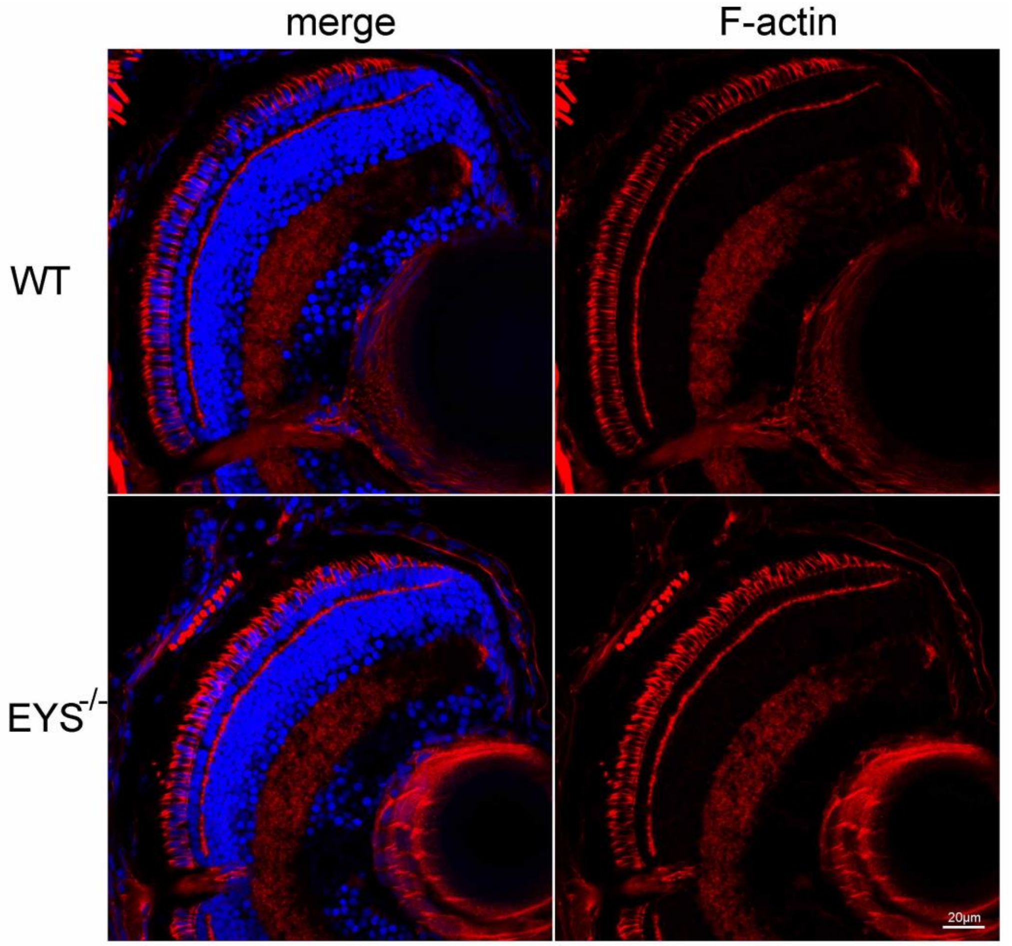

Supplementary Table 1

Table List of primary antibodies used in this study

Antibodies	Source	Antigen	Recognize	Dilution
Anti-rhodopsin	Made through Abclonal	Zebrafish rhodopsin	Zebrafish rhodopsin	1:500 for IF
Anti-opn1LM	Made through Abclonal	Zebrafish opn1LM	Zebrafish opn1LM	1:500 for IF
Anti-opn1MW	Made through Abclonal	Zebrafish opn1MW	Zebrafish opn1MW	1:500 for IF
Anti-opn1SW1	Made through Abclonal	Zebrafish opn1SW1	Zebrafish opn1SW1	1:500 for IF
Anti-opn1SW2	Abgent, Azb21565b	Zebrafish opn1SW2	Zebrafish opn1SW2	1：100 for IF
Anti-GNB3	Proteintech, 10081-1-AP	Human GNB3	Zebrafish GNB3	1:100 for IF<br>1:1000 for WB
Anti-PRPH2	Proteintech, 18109-1-AP	Human PRPH2	Zebrafish PRPH2	1:100 for IF<br>1:1000 for WB

Supplementary Table 2

Primers used for RT-qPCR.

Target gene	Forward (5’ to 3’)	Reverse (5’ to 3’)
grk1a	ctggttcctggacttccgtg	tttggtctggaaggcgtagg
grk1b	atcgagaagcgaatcctggc	gcaaatgctcaagcccacaa
grk7a	gcttatgacaccaagacccac	cgatccatttcgattccct
grk7b	tgcaggttgtcagaccttgg	accagtccactgaggtacga
pde6a	cagtcaacaagatcggggct	agctcaggtgaaacactcgg
pde6b	ggagcagccaccttactctg	gccaaagcccaatgatctgc
pde6c	acggtgcgtaagggctac	gatgcgctctttgtctggta
gcap1a	cgacatcaatggggatgggg	atggccacgatgtgtgtgag
recoverin	attcccaaagaggaccaaga	tcagccaatcgctcgttat
gnb1a	agatcagagatgcgcggaaag	caagtgtcccctcagtgtcc
gnb1b	actatcacagatcacagccaaca	gtgcatggcgtagattttagc
gnb3a	acgccattgggtttttcccca	ggacgtcacgccgcacatga
gnb3b	cacagattgaggcggctcgca	agttggacccgaggggctgg
