# Supplementary material for: Ablation of EYS in zebrafish causes mislocalisation of outer segment proteins, F-actin disruption and cone-rod dystrophy
Source: Sci Rep. 2017 Apr 5;7:46098. doi: 10.1038/srep46098 (PMC5380955; doi:10.1038/srep46098)
Supplement: Supplementary Information [file srep46098-s2.pdf]

Original data of western blot

Ablation of *EYS* in zebrafish causes mislocalisation of outer segment proteins, F-actin disruption and cone-rod dystrophy

Zhaojing Lu[1,+], Xuebin Hu[1,+], Fei Liu[1], Dinesh C. Soares[2], Xiliang Liu[1], Shanshan Yu[1], Meng Gao[1], Shanshan Han[1], Yayun Qin[1], Chang Li[1], Tao Jiang[1], Daji Luo[3], An-Yuan Guo[4], Zhaohui Tang[1*], Mugen Liu[1*]

PRPH2:

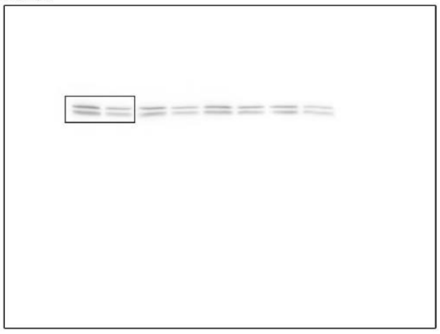

GNB3:

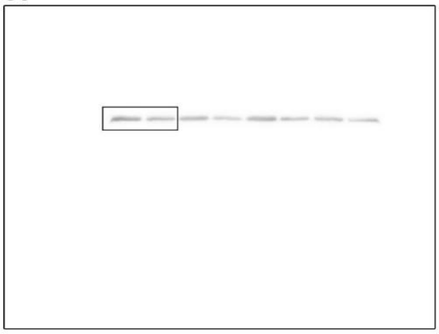

TUBULIN (PRPH2):

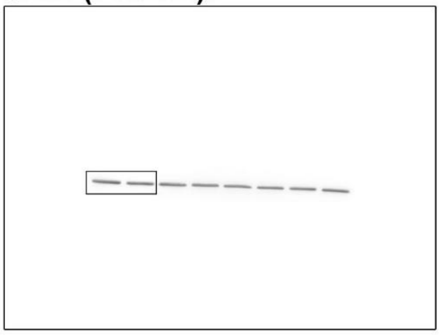

TUBULIN(GNB3):

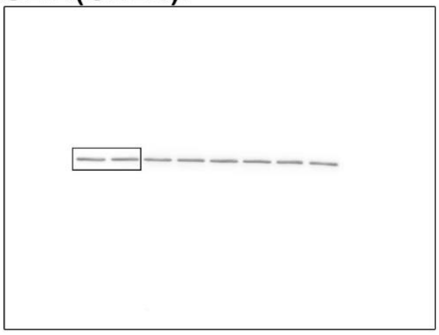

Protein levels of PRPH2 and GNB3 were detected by western blot at 6mpf. 1st, 3rd, 5th, 7th lanes are wild-type zebrafish; 2nd, 4th, 6th, 8th lanes are $eys^{-/-}$ zebrafish. Black box indicate the displayed gels/blots.
